# Analysis of provincial mortalities among agricultural vehicle users in East Azerbaijan, Iran (2006-2016)

**Published:** 2019-08

**Authors:** Homayoun Sadeghi-Bazargani, Bahram Samadirad, Mina Golestani

**Affiliations:** ^*a*^Road Traffic Injury Research Center, Statistics and Epidemiology Department, Tabriz University of Medical Sciences, Tabriz, Iran.; ^*b*^Legal Medicine Research Center, Legal Medicine Organization, Tehran, Iran.

## Abstract

**Background:**

Agricultural industry is one of the most hazardous industry. The combination of causes in the agricultural industry determines the potential to damage. Objective: In the current paper, we provide the latest epidemiological data on the agriculture fatalities, over a decade of time between April 2006 and April 2016, in East Azerbaijan province, Iran.

**Methods:**

In this cross-sectional study, data extracted from AVU fatalities in the East Azerbaijan Forensic Medicine Organization database, for a decade assessment between March 2006 and March 2016. Data analysis was conducted through STATA 13 statistical software (STATA Corp, Texas). Descriptive statistics were calculated such as mean, frequency, relative frequency, standard deviation (SD), 95% confidence intervals (95% CI).

**Results:**

A total of 9435 fatal traffic injury cases were registered in the database through the Iranian Shamsi calendar years of 1385 - 1394 (March 2006 and March 2016). Rollover was the most common mechanism of accidents, comprising 86% of the cases. Head trauma was the leading factor of death, accounting for 51.3% of the cases (39 victims). More common as the main cause of death was bleeding among those who died prior either at the accident site or while being transferred to the hospital (P = 0.01).

**Conclusions:**

According to the current report, most of the victims have the driver role that it seems that related to the lack of proper training in the driving, as well as non-use of personal protective equipment and lack of appropriate skills in driving in addition to low literacy rate and the presence of younger AVU. A supplementary program to renovating old agricultural machinery in using or improving safety through the addition of protective equipment and structures can help reduce AVU mortalities.

**Keywords:**

Mortality, Agriculture, Vehicle, Road traffic user

